# Epidermal growth factor receptor (EGFR) gene promoter methylation and cetuximab treatment in colorectal cancer patients

**DOI:** 10.1038/bjc.2011.161

**Published:** 2011-05-10

**Authors:** M Scartozzi, I Bearzi, A Mandolesi, R Giampieri, L Faloppi, E Galizia, F Loupakis, A Zaniboni, F Zorzi, T Biscotti, R Labianca, A Falcone, S Cascinu

**Affiliations:** 1 Department of Clinica di Oncologia Medica, AO Ospedali Riuniti-Università Politecnica delle Marche, Via Conca, 60020 Ancona, Italy; 2 Department of Anatomia Patologica, AO Ospedali Riuniti-Università Politecnica delle Marche, Ancona, Italy; 3 Department of Oncologia Medica, Ospedale ‘Profili’, Fabriano, Italy; 4 Department of Oncologia Medica, Università degli Studi di Pisa, Pisa, Italy; 5 Department of Oncologia Medica, Fondazione Poliambulanza, Brescia, Italy; 6 Department of Anatomia Patologica, Fondazione Poliambulanza, Brescia, Italy; 7 Department of Oncologia Medica, Ospedali Riuniti, Bergamo, Italy

**Keywords:** EGFR promoter methylation, cetuximab, colorectal cancer

## Abstract

**Background:**

Epidermal growth factor receptor (EGFR) promoter methylation may be responsible for the loss of EGFR expression in neoplastic cells. The primary aim of our study was to verify a possible correlation between *EGFR* gene promoter methylation and clinical outcome in metastatic colorectal cancer patients receiving chemotherapy with irinotecan and cetuximab.

**Methods:**

Colorectal samples from patients treated with irinotecan–cetuximab were analysed for EGFR promoter methylation and EGFR immunohistochemistry.

**Results:**

Fifty-two patients were analysed. Thirty patients (58%) showed *EGFR* promoter hypermethylation. In *EGFR* promoter methylated and *EGFR* promoter unmethylated patients, we observed a partial response in 3 (10%) and 13 (59%) patients, respectively (*P*=0.03), progressive disease was obtained in 19 (63%) and 2 (9%) patients, respectively, with *EGFR* promoter methylated and *EGFR* promoter unmethylated tumours (*P*=0.0001). Median progression-free survival was 2.4 months in patients showing *EGFR* promoter methylated tumours and 7.4 months for those who had *EGFR* promoter unmethylated tumours (*P*<0.0001; Figure 1). Median overall survival was 6.1 months in patients showing *EGFR* promoter methylated tumours and 17.8 months for those who had *EGFR* promoter unmethylated tumours (*P*<0.0001; Figure 2). CONCLUSION: *EGFR* promoter hypermethylation, after confirmation in larger data set, may represent a valuable asset in further studies investigating EGFR as a therapeutic target in colorectal cancer.

The molecular mechanisms underlying response or resistance of epidermal growth factor receptor (EGFR) overexpressing colorectal tumours to anti-EGFR compounds are still largely unknown. However, economic costs and toxicity risks deriving from the use of anti-EGFR therapeutic options made increasingly essential the identification of molecular or clinical predictive factors of response (or resistance) for a better, more accurate, actually targeted, selection of patients more likely to benefit from such a treatment approach. The main research areas in this setting have been focusing on the role of EGFR protein expression, *EGFR* gene amplification, *EGFR* mutations, and markers of EGFR downstream signalling (Moroni *et al*, 2005; Benvenuti *et al*, 2007; Scartozzi *et al*, 2007, 2009; Di Nicolantonio *et al*, 2008; Ng and Zhu, 2008; Loupakis *et al*, 2009; Perrone *et al*, 2009). Only after several years of intense translational research and clinical absence of predictive factors, the introduction of the K-RAS mutational status seemed to possess the necessary potential for a full translation into clinical practice of the concept of targeted therapy in this setting (Di Fiore *et al*, 2007; Lievre *et al*, 2008; Van Cutsem *et al*, 2009).

However, if on the one hand we are now able to exclude from anti-EGFR treatment patients with putative refractory colorectal tumours (i.e., those harbouring a K-RAS mutant status), on the other hand we are still incapable to accurately select responding patients among those without K-RAS mutations. In fact clinical observations suggested that a non-negligible proportion of patients, usually ranging from 40 to 70%, does not seem to benefit from the use of anti-EGFR-targeted antibodies although in the absence of a mutation of the *K-RAS* gene (i.e., K-RAS wild-type patients) (Di Fiore *et al*, 2007; Jonker *et al*, 2007; Van Cutsem *et al*, 2007, 2009; Lievre *et al*, 2008).

We now know that under normal circumstances, EGFR expression is primarily regulated by the abundance of its m-RNA (Scartozzi *et al*, 2007; Van Cutsem *et al*, 2009). This observation is of particular relevance if we consider that EGFR m-RNA expression demonstrated a possible correlation with survival during anti-EGFR treatment (Xu *et al*, 1984; Merlino *et al*, 1985; Vallböhmer *et al*, 2005). At least hypothetically, EGFR promoter silencing may then affect clinical outcome of patients treated with anti-EGFR strategies through the inhibition of EGFR m-RNA expression.

Cytosine methylation of promoter-associated CpG islands is an important epigenetic mechanism of gene silencing that is frequently observed in cancer, leading to inhibition of gene transcription (Xu *et al*, 1984; Merlino *et al*, 1985). Hypermethylation typically affects tumour-suppressor genes, but can also silence oncogenes such as *COX219* and *TERT (*Devereux *et al*, 1999; Santini *et al*, 2001; Jones and Baylin, 2002).

Montero *et al* analysed the presence of *EGFR* promoter hypermethylation in a series of cell lines and tissues, suggesting that *EGFR* promoter hypermethylation may represent a relevant event in breast, head and neck, and lung tumours. In this study, *EGFR* hypermethylation was observed in none of the 17 colorectal tumours tested and in 7 of the 17 (24%) normal colon tissue (Montero *et al*, 2006). However, in a larger analysis including 63 colorectal tumours we previously demonstrated that *EGFR* promoter methylation should not be considered a rare event in colorectal tumours as this biological phenomenon occurred in as many as 39% of all cases analysed (Scartozzi *et al*, 2009).

Based on these considerations, we then hypothesised that *EGFR* promoter methylation may be responsible for the loss of EGFR expression in neoplastic cells, with the consequent loss of the therapeutic target for anti-EGFR monoclonal antibodies. These observations may be relevant for clinical outcome prediction with the use of anti-EGFR treatment strategies and could also indicate new research perspectives for the introduction of pharmacological agents able to determine re-expression of the therapeutic target (EGFR) in this area. The aim of our study was then to verify a possible correlation between *EGFR* gene promoter methylation and clinical outcome in metastatic colorectal cancer patients receiving chemotherapy with irinotecan and cetuximab. The possible correlation between *EGFR* promoter methylation status and EGFR protein expression was also tested.

## Patients and methods

### Patients selection

Patients with histologically proven EGFR-positive, K-RAS wild-type, metastatic, colorectal cancer receiving a combination of cetuximab and irinotecan after at least one line of previous chemotherapy were eligible for our analysis. To be eligible, patients must also have received an irinotecan-based chemotherapy regimen for at least 6 weeks and must have presented progression of disease during receipt of this regimen or within 3 months thereafter. All patients received cetuximab at an initial dose of 400 mg per square metre followed by weekly infusions of 250 mg per square metre. Irinotecan was administered at a dose of 180 mg per square metre every 2 weeks either alone or in combination with five fluorouracil and leucovorin. Tumour response was evaluated every 8 weeks by clinicians’ assessment and according to the Response Evaluation Criteria in Solid Tumours (RECIST).

Formalin-fixed and paraffin-embedded tumour samples (either primary site or metastasis or both when available) of colorectal cancer patients were analysed for EGFR protein expression (immunohistochemistry) and for EGFR promoter methylation.

### EGFR promoter methylation study

Analysis of EGFR promoter methylation was performed following a DNA Extraction Protocol from paraffin-embedded tissue and a methylation-specific PCR (MSP). The tumour samples were processed according to the QIAamp DNA mini Tissue Protocol, using QIAamp DNA Mini Kit (QIAGEN GmbH, Hilden, Germany). Before PCR amplification, the DNA extract was treated with sodium bisulphite as described in the handbook of the ‘EpiTect Bisulfite Kit’ (QIAGEN GmbH). Bisulphite modification of DNA to convert all unmethylated cytosines to uracil and then to thymidine during the subsequent PCR step while leaving the methylated cytosines unaffected was performed as described by Herman *et al* (1996). For PCR amplification, two sets of primers were designed from nt −130 to −300 (relative to ATG) in the 5′-untranslated region of the human EGFR promoter.

The primer sequences used were 5′-TGTTTTGTTTTTTTGTGTTTTGGTTTGTGT-3′ (sense) and 5′-CATCCAATCTAAACAACAACAACCACCA-3′ (antisense) for unmethylated DNA and 5′-TGTTTTTTCGCGTTTCGGTTCGCGC-3′ (sense) and 5′-CGTCTAAACGACGACGACCGCCG-3′(antisense) for methylated DNA, both of which amplify ∼150 bp products (Nagothu *et al*, 2004). The PCR mixture contained 1 × PCR buffer, Minus Mg; 0.2 mM dNTP mixture (each); 1.5 mM MgCl_2_; 0.2 *μ*M Primer mix (each); 1.0 unit Platinum *Taq* DNA Polymerase (Invitrogen, Carlsbad, CA, USA); and bisulphite-modified DNA (of 1 ng–2 *μ*g) in a final volume of 50 *μ*l. Controls without DNA were performed for each set of PCRs. Each PCR product (30 *μ*l) was directly visualised on 10% acrylamide gels. The gel was stained with ethidium bromide and photographed under UV illumination. An enzymatically methylated human male genomic DNA (CpGenome Universal Methylated DNA CHEMICON International) was used as a methylation-positive control for gene methylation studies and was processed as above mentioned.

### Immunohistochemistry

Epidermal growth factor receptor (DakoCytomation, Carpinteria, CA, USA) was evaluated with an immunohistochemistry technique on 5 *μ*m-thick tissue section obtained from paraffin-embedded specimens fixed in 10% (v/v) neutral buffered formalin, and was performed using kit EGFR PharmaDx (DakoCytomation) according to the manufacturer's instructions as previously described (Scartozzi *et al*, 2004). Briefly, the intensity of EGFR reactivity was scored using a three-tier system as follows: 1+ (weak intensity: faint brown membranous staining); 2+ (moderate intensity: brown membranous staining of intermediate darkness producing a complete or incomplete circular outline of the neoplastic cell); 3+ (strong intensity: dark brown or black membranous staining producing a thick outline, complete or incomplete of the neoplastic cell) (Scartozzi *et al*, 2004).

### Statistical analysis

Statistical analysis was performed with MedCalc package (MedCalc v9.4.2.0, MedCalc Software, Mariakerke, Belgium).

The association between categorical variables was analysed by *χ*^2^-test. Survival distribution was estimated by the Kaplan–Meier method. Significant differences in probability of relapsing between the strata were evaluated by log-rank test.

A significant level of 0.05 was chosen to assess the statistical significance.

For statistical analysis, overall survival (OS) and progression-free survival (PFS) were defined, respectively, as the interval between the start of cetuximab and irinotecan therapy to death or last follow-up visit and as the interval between the start of cetuximab and irinotecan therapy to clinical progression or death or last follow-up visit if not progressed.

## Results

Fifty-two metastatic colorectal cancer patients were eligible for our analysis: 33 patients were males (63%) and 19 females (37%), median age at diagnosis was 62 years (range 35–80) (Table 1).

In 27 patients (52%), the EGFR promoter methylation study was conducted on primary colorectal tumours, in 21 cases (40%) both primary tumours and corresponding metastasis (liver metastases in all cases) were available for analysis, while in 4 cases (8%) only the metastatic site (liver metastases in all cases) was investigated. For study purposes when results for EGFR methylation status in primary colorectal tumours and corresponding metastases resulted conflicting, only the methylation status in metastases was considered biologically relevant. Although data guiding this choice are lacking the purpose of the study was to assess the correlation between *EGFR* gene promoter methylation and clinical outcome in patients treated with cetuximab for metastatic disease. Therefore, we can hypothesise that the methylation status in metastases is more relevant for response/resistance to such treatment approach.

Globally, 30 patients (58%) showed *EGFR* promoter hypermethylation either in primary colorectal cancer or in metastasis. In 12 cases (40%), *EGFR* promoter methylation resulted biallelic, whereas in the remaining 18 tumours (60%) only one allele resulted methylated. This two groups of patients (i.e., those with monoallelic EGFR promoter methylation and those with monoallelic *EGFR* promoter methylation resulted) comparable for all main clinical characteristics and experienced a similar clinical outcome during treatment with cetuximab (Table 2). Among the 21 patients in whom both primary site and metastases were available for *EGFR* methylation study, *EGFR* methylation status of primary tumour was in accordance with that of metastasis in 16 patients (76%): 4 patients with *EGFR* promoter hypermethylation and 12 patients with unmethylated tumours. In the remaining five patients (24%), there was not concordance in *EGFR* promoter methylation status between primary tumour and metastasis. In particular, two patients with *EGFR* hypermethylation in the primary tumour showed unmethylated *EGFR* in metastasis and three metastases showing *EGFR* promoter hypermethylation derived from unmethylated EGFR primary tumours.

**Figure 1:**
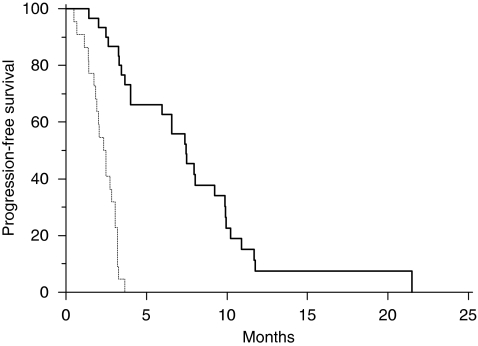
Kaplan–Meier curves for median progression-free survival (PFS) of colorectal cancer patients treated with irinotecan and cetuximab with *EGFR* promoter methylated 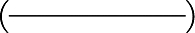 and without *EGFR* promoter methylated 
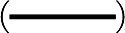
 tumours (2.4 *vs* 7.4 months, *P*<0.0001).

**Figure 2:**
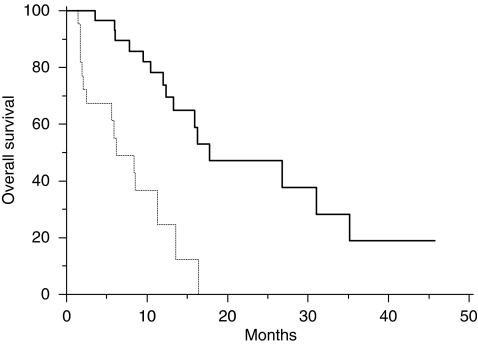
Kaplan–Meier curves for median overall survival (OS) of colorectal cancer patients treated with irinotecan and cetuximab with *EGFR* promoter methylated 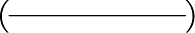 and without *EGFR* promoter methylated 
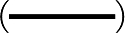
 tumours (6.1 *vs* 17.8 months, *P*<0.0001).

All major clinical characteristics resulted comparable among *EGFR* promoter methylated and *EGFR* promoter unmethylated groups of patients. In particular, no differences were noticed for sex, age at diagnosis, and previous lines of chemotherapy (Table 1). On the contrary in *EGFR* promoter methylated and *EGFR* promoter unmethylated patients, we observed a partial response in 3 (10%) and 13 (59%) patients, respectively (*P*=0.03), progressive disease was obtained in 19 (63%) and 2 (9%) patients, respectively, with *EGFR* promoter methylated and *EGFR* promoter unmethylated tumours (*P*=0.0001). No statistically significant differences were noticed for stable disease (Table 1). Median PFS was 2.4 months in patients showing *EGFR* promoter methylated tumours and 7.4 months for those who had *EGFR* promoter unmethylated tumours (*P*<0.0001; Figure 1). Median OS was 6.1 months in patients showing *EGFR* promoter methylated tumours and 17.8 months for those who had *EGFR* promoter unmethylated tumours (*P*<0.0001; Figure 2).

Overall, EGFR immunohistochemical assessment resulted positive in 36 patients (69%).

Lack of EGFR protein expression was observed in nine *EGFR* promoter methylated tumours (30%) and in seven *EGFR* promoter unmethylated tumours (32%).

## Discussion

The expanding role of anti-EGFR therapeutic modalities for the treatment of colorectal cancer patients, along with the growing number of cases potentially requiring such a treatment approach, made the need for a correct and reliable identification of responding tumours increasingly crucial.

Unfortunately beside K-RAS mutational status and although the intense ongoing translational research no clear indications for further predictive molecular markers are available at the moment.

Transcriptional silencing of tumour-suppressor genes, by methylation of CpG dinucleotide-rich areas in gene promoter, is one of the major epigenetic mechanisms leading to inactivation (and thus to lack of m-RNA expression) of important growth control genes (Herman and Baylin, 2003). However, data about *EGFR* gene silencing by promoter methylation are substantially lacking and may help clarifying the role of this epigenetic mechanism on colorectal cancer biology (Scartozzi *et al*, 2009). The implications for anti-EGFR treatment options are also relevant. Loss of *EGFR* expression consequent to promoter methylation may underlie loss of the therapeutic target and may then indicate both a predictive factor for anti-EGFR therapy and new research perspectives for the use of demethylating agents.

In our analysis, *EGFR* promoter methylation was evident in 30 patients (58%), thus indicating that this biological phenomenon should not be considered an infrequent event in colorectal tumours and confirming our previous report. Moreover, patients presenting an *EGFR* promoter methylated tumour experienced in fact a worse clinical outcome thus confirming our hypothesis of a role for *EGFR* promoter methylation in determining the efficacy of anti-EGFR-targeted monoclonal antibodies.

A clear correspondence between *EGFR* promoter methylation and loss of EGFR immunohistochemical expression was not evident. It is then unlikely that loss of EGFR expression as determined with immunohistochemistry may be related to *EGFR* promoter hypermethylation. When we consider that EGFR promoter methylation represents a predictive factor for cetuximab treatment in our series, the observation of a lack of concordance of this biological phenomenon with EGFR immunohistochemistry expression seems clinically sound and in accordance to previous data, indicating that loss of EGFR immunohistochemical expression should not be considered a predictive factor for anti-EGFR monoclonal antibodies activity (Cunningham *et al*, 2004). However, it is also likely that immunohistochemistry evaluation of EGFR protein expression may not be accurate enough to detect loss of EGFR protein in cancer tissues, thus compromising data analysis and interpretation in this setting (Atkins *et al*, 2004).

It is also important to note that only in 12 patients (40%) *EGFR* methylation resulted biallelic, and methylation of only one allele could not be sufficient to actually silence the gene, inducing a total loss of protein production. On the other hand, EGFR promoter methylation of one allele seemed able to determine resistance to anti-EGFR therapy in our series and we could not find substantial difference among the two groups of patients with EGFR promoter methylation (i.e., those with monoallelic EGFR promoter methylation and those with monoallelic EGFR promoter methylation). Therefore, we can speculate that other relevant biological mechanisms, such as loss of heterozygosity, may contribute to loss of gene function along with monoallelic promoter methylation in tumour tissues.

However, the relatively small number of patients analysed in our series represents a clear limitation for a definitive conclusion about the role of EGFR promoter methylation in determining response to anti-EGFR treatments, and our findings should be considered speculative. A further possible limiting factor is represented by the proportion of patients with a concordant *EGFR* methylation status between primary tumour and metastases (76%). This observation is however in line with our previous observation of a concordance for *EGFR* methylation status in 69% of patients with metastatic colorectal cancer.

The possible role of *EGFR* promoter methylation in *EGFR* gene silencing could provide new clues in anti-EGFR biological agents use optimisation. It has been in fact reported that treatment with the demethylating agent decitabine may result in the re-expression of EGFR in two breast cancer cell lines, in which *EGFR* transcription was abolished by *EGFR* promoter hypermethylation. Both cell lines are relatively resistant to killing by the EGFR inhibitor gefitinib; however, after co-treatment with decitabine and gefitinib, a significant effect on the induction of apoptosis was observed (Montero *et al*, 2006).

Combined approaches targeting *EGFR* dysfunction may be useful for patients with *EGFR* methylated tumours and could represent the basis for prospective studies aiming to compare clinical response with EGFR directed therapeutic agents. Strategies have been developed that combine treatments with drugs reactivating silenced gene expression (such as demethylating drugs) with secondary agents that target the re-expressed genes and/or reconstituted signal transduction pathways (Karpf and Jones, 2002). This treatment approach looks appealing for further dedicated trials in *EGFR* promoter methylated tumours.

We believe that *EGFR* promoter hypermethylation, after confirmation in larger data set, may represent a valuable and important asset to be considered in further studies investigating the role of EGFR as a therapeutic target in colorectal cancer patients.

## Figures and Tables

**Table 1:** Patients characteristics and main study results

	**Whole group (*n*=52)**	**EGFR unmet (*n*=22, 42%)**	**EGFR met (*n*=30, 58%)**	***P*-value**
Age (range)	62 (35–80)	63 (35–78)	62 (37–80)	
				
*Sex*				
Males	33 (63%)	15 (68%)	18 (66%)	
Females	19 (37%)	7 (32%)	12 (34%)	
				
*Previous lines of treatment*				
1	6 (12%)	4 (18%)	2 (7%)	
2–3	46 (88%)	18 (82%)	28 (93%)	
				
*Treatment*				
mFOLFIRI+cetuximab	18 (35%)	8 (36%)	10 (33%)	
Irinotecan+cetuximab	34 (65%)	14 (68%)	20 (67%)	
				
*Response rate*				
PR	16 (31%)	13 (59%)	3 (10%)	0.03
SD	15 (29%)	7 (32%)	8 (27%)	
PD	21 (40%)	2 (9%)	19 (63%)	0.0001
				
Median PFS (months)	3.2	7.4	2.4	<0.0001
Median OS (months)	13.3	17.8	6.1	<0.0001

Abbreviations: mFOLFIRI=modified FOLFIRI (irinotecan 180 mg sqm^−1^ d1, 5FU bolus 400 mg sqm^−1^ d1, 5FU 2400 mg sqm^−1^ continuous infusion for 46 h); PR=partial remission; SD=stable disease; PD=progressive disease; PFS=progression-free survival; OS=overall survival; EGFR=epidermal growth factor receptor.

Only statistically significant *P*-values have been indicated.

**Table 2:** Patients characteristics and main results according to *EGFR* promoter methylation status (monoallelic *vs* biallelic EGFR promoter methylation)

	**EGFR monoallelic (*n*=18, 60%)**	**EGFR biallelic (*n*=12, 40%)**	***P*-value**
Age (range)	61 (35–78)	62 (37–80)	
			
*Sex*			
Males	11 (61%)	7 (58%)	
Females	7 (39%)	5 (42%)	
			
*Previous lines of treatment*			
1	1 (5%)	1 (8%)	
2–3	17 (95%)	11 (92%)	
			
*Treatment*			
mFOLFIRI+cetuximab	6 (33%)	4 (33%)	
Irinotecan+cetuximab	12 (66%)	8 (66%)	
			
*Response rate*			
PR	2 (11%)	1(8%)	Ns
SD	5 (28%)	3 (25%)	Ns
PD	11 (61%)	8 (67%)	Ns
			
Median PFS (months)	2.2	2.4	Ns
Median OS (months)	5.9	6.1	Ns

Abbreviations: mFOLFIRI=modified FOLFIRI (irinotecan 180 mg sqm^−1^ d1, 5FU bolus 400 mg sqm^−1^ d1, 5FU 2400 mg sqm^−1^ continuous infusion for 46 h); PR=partial remission; SD=stable disease; PD=progressive disease; PFS=progression-free survival; OS=overall survival; EGFR=epidermal growth factor receptor.

No statistically significant differences could be noticed among the two groups of patients.
